# Development of an Electrochemical Paper-Based Device Modified with Functionalized Biochar for the Screening of Paracetamol in Substandard Medicines

**DOI:** 10.3390/molecules29225468

**Published:** 2024-11-20

**Authors:** Martin Kassio Leme da Silva, Francisco Contini Barreto, Guilherme dos Santos Sousa, Rafael Plana Simões, Gaurav Ahuja, Samriddha Dutta, Ashok Mulchandani, Ivana Cesarino

**Affiliations:** 1 School of Agriculture, São Paulo State University (UNESP), Botucatu 18610-034, SP, Brazil; martin.leme@unesp.br (M.K.L.d.S.); francisco.c.barreto@unesp.br (F.C.B.); gs.sousa@unesp.br (G.d.S.S.); rafael.simoes@unesp.br (R.P.S.); 2 Department of Chemical and Environmental Engineering, University of California, Riverside, CA 92521, USA; gaurav.ahuja001@email.ucr.edu (G.A.); adani@engr.ucr.edu (A.M.); 3 Department of Bioengineering, University of California, Riverside, CA 92521, USA; sdutt013@ucr.edu

**Keywords:** electrochemical paper-based analytical devices (ePADs), 3D printing, conductive ink, substandard drugs, paracetamol

## Abstract

The global prevalence of counterfeit and low-quality pharmaceuticals poses significant health risks and challenges in medical treatments, creating a need for rapid and reliable drug screening technologies. This study introduces a cost-effective electrochemical paper-based device (ePAD) modified with functionalized bamboo-derived biochar (BCF) for the detection of paracetamol in substandard medicines. The sensor was fabricated using a custom 3D-printed stencil in PLA, designed for efficient production, and a 60:40 (m/m) graphite (GR) and glass varnish (GV) conductive ink, resulting in a robust and sensitive platform. The electroactive area of the ePAD/BCF sensor was determined as 0.37 cm^2^. Characterization via SEM and cyclic voltammetry (CV) verified its structural and electrochemical stability. The sensor demonstrated linear detection of paracetamol from 5.0 to 60.0 µmol L^−1^ with a detection limit of 3.50 µmol L^−1^. Interference studies showed high selectivity, with recoveries of over 90%, and the sensor successfully quantified paracetamol in commercial analgesic and anti-flu samples. This sustainable, bamboo-based ePAD offers a promising solution for rapid on-site pharmaceutical quality control, with significant potential to enhance drug screening accuracy.

## 1. Introduction

Substandard drugs are a serious worldwide issue that generates grave global health, economic and social consequences, requiring innovative and safe anti-counterfeiting solutions [1,2]. The World Health Organization (WHO) classifies these drugs as products that do not meet the quality standards established by regulatory authorities and that are incorrectly labeled regarding identity and/or origin [3,4,5]. Counterfeit drugs may contain the correct ingredients, the incorrect ingredients, the active pharmaceutical ingredient in the incorrect concentration, no active pharmaceutical ingredient or false labeling [3], and these drugs are the cause of approximately 100,000 deaths per year worldwide [6]. In a global context, researchers estimate that 1 in 10 pharmaceutical products are substandard [5,6,7], and this number could reach 3 in 10 in some regions of Africa, Asia and Latin America [6]. Among the most counterfeited pharmaceuticals, it is estimated that 12% are painkillers [1].

Acetaminophen, popularly known as paracetamol (PARA), is an analgesic, antipyretic and non-steroidal anti-inflammatory that can be purchased without a prescription [7,8]. Paracetamol is used to treat fever, headaches, muscle pain, body aches, toothaches, arthritis and other problems [8,9], and its consumption is estimated at 30 million doses per day [9]. Paracetamol is among the most counterfeited medicines [10], and the high consumption associated with the counterfeiting rate can cause several problems, which necessitates the development of analytical tools to monitor medicines that contain this substance [8]. In doses above the permitted limit, this drug can cause various illnesses, from convulsions, nausea, insomnia and tremors to kidney failure, liver necrosis and death [7,9,11].

It can be difficult to discern substandard drugs from authentic products just on visual inspection, making their identification and analysis a complex and challenging task [1]. Since analytical techniques can offer a trustworthy and objective approach to identify and quantify the active ingredients, contaminants, and other components in a sample, they are consequently essential for the detection and analysis of these pharmaceutical products. There are many analytical techniques that can be used for the detection and analysis of substandard drugs, including high-performance liquid chromatography (HPLC) [5], mass spectrometry (MS) [1], benchtop NMR spectroscopy [12], near-infrared chemical imaging (NIR-CI) [13], thin layer chromatography (TLC) [1], Raman spectroscopy [10] and UV spectrophotometry [5]. 

The use of electrochemical sensors to identify substandard drugs has gained popularity in recent years [3,4]. They are able to detect a variety of substances, such as drugs [14,15], pesticides [16,17] and biomolecules [18,19], and are highly sensitive and selective. Due to their portability and simplicity of use, they can be especially useful in situations that require in situ analysis [20]. Additionally, real-time data can be obtained from electrochemical sensors [18,20], and detection in complicated matrices such as blood [21] or urine [3] samples can be carried out.

The efficacy of electrochemical sensors has been demonstrated through several scientific investigations. These studies have used a variety of materials to modify electrodes, such as graphene oxide [14] and carbon nanotubes [22], and, more recently, materials based on renewable carbon, such as biochar [23] and hydrochar [24], have gained considerable prominence. These materials are obtained from biomass pyrolysis and can be used to produce cheaper and more sustainable sensors [23,24]. Their effectiveness for determining compounds is also being proven, which makes them a great option for manufacturing analytical devices [23,24,25,26].

The emergence of electrochemical paper-based analytical devices (ePADs) presents an opportunity to develop cost-effective tools for identifying potential water contamination. This technology has been utilized in electroanalysis due to its favorable characteristics, such as high sensitivity, versatility in applications, the ability to produce different electrode configurations, and suitability for on-site monitoring [27,28]. Typically, an ePAD and a screen-printed electrode (SPE) consist of three electrodes printed with carbon paste or ink [29]. To enhance the durability of the printing process, the ink is primarily composed of conductive materials like graphite or carbon black, along with a binder or plasticizer [30]. On a laboratory scale, carbon-based inks can be created using graphite powder and traditional binders such as glass varnish [28].

Therefore, this work aims to develop a paper-based analytical device (ePAD) using a 3D-printed Polylactic Acid (PLA) stencil with the creation of a hydrophobic barrier through solid wax printing. Furthermore, functionalized bamboo-based biochar (BCF) was used as a modifier to make a sustainable and affordable sensor for detecting paracetamol in low-quality or counterfeit medicines.

## 2. Results

### 2.1. Morphological Characterization

**Figure 1:**
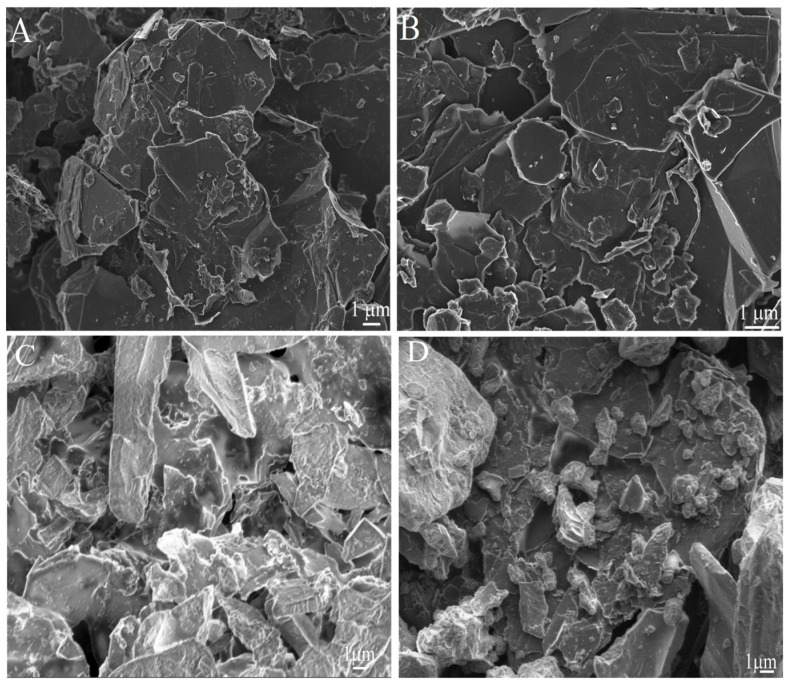
SEM images of carbon ink spread on a paper substrate at magnifications of (**A**) 5000× and (**B**) 10,000×; (**C**,**D**) ePAD modified with BCF.

Scanning electron microscopy (SEM) images of the fabricated ePADs are shown in Figure 1, providing insights into the morphological characteristics that contribute to the sensor’s performance. In Figure 1A, cellulose fibers are visible, interspersed with graphite structures, indicative of the paper substrate’s natural texture combined with the conductive ink. This fiber–graphite interaction suggests good adhesion and integration of the ink with the paper substrate, which is essential for consistent electrochemical behavior.

Figure 1B reveals the graphite structures as randomly arranged agglomerates, with particle sizes ranging from 0.5 to 50 µm. This irregular surface profile likely results from the use of a brush in the manual printing technique developed in this study, which introduces a degree of heterogeneity in particle distribution. Such roughness can be advantageous, as it increases the effective surface area available for electrochemical reactions, potentially enhancing sensitivity.

Figure 1C,D display the morphology of the ePAD after modification with the BCF material. The presence of biochar is evident, with the particles forming a heterogeneous layer characterized by a variety of sizes and the presence of porous, honeycomb-like structures [31]. These pores are beneficial as they can facilitate electrolyte access, improving ion exchange and enhancing electron transfer kinetics on the sensor surface. The biochar’s porous nature, combined with its irregular distribution, contributes to an increased surface area and additional active sites, which are crucial for improving the sensor’s responsiveness to an analyte [32]. Overall, the SEM images suggest that the integration of BCF creates a more complex surface morphology, which can promote greater electrochemical activity and improve detection sensitivity.

### 2.2. Electrochemical Characterization of ePADs

**Figure 2:**
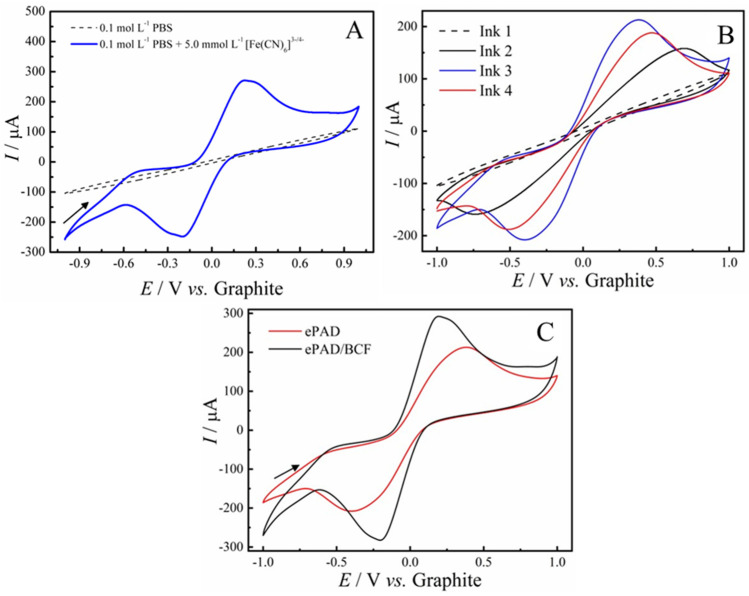
Cyclic voltammograms in PBS 0.2 mol L^−1^ containing 0.1 mol L^−1^ of KCl and 5.0 mmol L^−1^ of [Fe(CN)_6_]^3−/4−^: (**A**) Comparison of ePAD performances with different formulations of GR-GV conductive ink. (**B**) Cyclic voltammograms of ePADs fabricated with different conductive ink compositions (Ink 1: 80:20, Ink 2: 70:30, Ink 3: 60:40, and Ink 4: 50:50 GR ratios, m/m) in 0.1 mol L^−1^ PBS containing 5.0 mmol L^−1^ [Fe(CN)_6_]^3−^/^4−^ at 50 mV s^−1^. (**C**) Voltammetric response of the ePAD sensor without modification (red line) and after BCF modification (black line).

To determine the optimal composition of the conductive ink for manufacturing ePAD sensors, the ratio between graphite (GR) and glass varnish (GV) was optimized. Figure 2A shows the voltammetric response of the ePAD sensor in 0.2 mol L^−1^ PBS solution (dashed line) and in the presence of 5.0 mmol L^−1^ of the redox couple [Fe(CN)_6_]^3−/4−^ (solid line) at a scan rate of 50 mV s^−1^. The results showed no redox process in the absence of the probe, indicating that the observed oxidation and reduction processes are associated with the iron species in the solution.

Figure 2B presents cyclic voltammograms obtained with ePADs manufactured with different GR-GV ink compositions, using a 150 µL aliquot of a 0.2 mol L^−1^ PBS solution (pH 7.4) containing 0.1 mol L^−1^ KCl and 5.0 mmol L^−1^ of the redox couple [Fe(CN)_6_]^3−/4−^ at a scan rate of 50 mV s^−1^. The GR-GV (m/m) ratios were 80:20 (ink 1), 70:30 (ink 2), 60:40 (ink 3), and 50:50 (ink 4), based on a total mass of 150 mg. Table 1 presents the anodic (*I*_pa_) and cathodic (*I*_pc_) peak currents, as well as the separation of anodic and cathodic peak potentials (Δ*E*_p_) for the probe’s redox potentials across all sensors evaluated. As illustrated, the ePAD sensor manufactured with the GR-GV ink at a 60:40 ratio (ink 3) demonstrated the best voltammetric response, with an oxidation current of *I*_pa_ = 213 µA, a reduction current of *I*_pc_ = −208 µA, and a peak potential separation (Δ*E*_p_) of 772 mV vs. graphite (pseudo-reference). Additionally, this ink showed superior handling properties during sensor fabrication, without cracking or leakage through the PLA stencil. Therefore, this ink was selected for further investigation. Although inks with higher conductive material content exist, excessive amounts can lead to the blockage of active sites, thereby reducing the material’s sensitivity [33].

After the modification process with 10 µL of BCF (1.0 mg/mL) on the working electrode, we can observe that the voltammogram showed a better reversibility of the redox process, with Δ*E*_p_ = 387 mV vs. graphite (Figure 2C), along with a significant increase in the anodic and cathodic current responses of the redox probe (Table 1). The increase in anodic and cathodic currents may be attributed to the acid treatment of biochar, which results in a reduction in both the H/C and O/C ratios. A lower H/C ratio suggests increased carbonization and the formation of more aromatic structures within the material. Additionally, the decrease in the O/C ratio indicates a reduction in hydrophilicity and polarity, altering the material’s surface properties. Furthermore, biochar functionalization likely contributes to the formation of new active sites on the surface, enhancing the material’s electrochemical reactivity [34,35].

**Figure 3:**
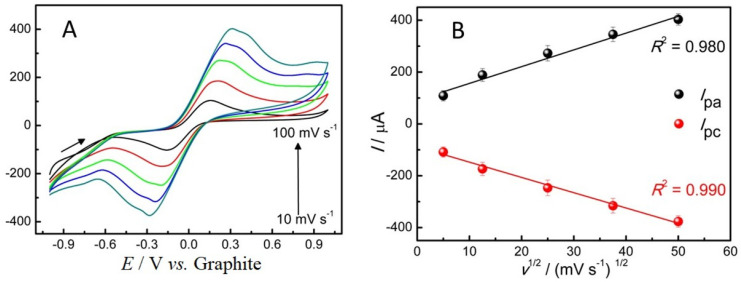
(**A**) Cyclic voltammograms obtained with the ePAD/BCF sensor in a 0.2 mol L^−1^ PBS solution (pH 7.4) containing 0.1 mol L^−1^ of KCl and 5.0 mmol L^−1^ of the redox couple [Fe(CN)_6_]^3−/4−^ for scanning speeds of 10, 25, 50, 75 and 100 mV s^−1^. (**B**) Graph of *I* vs. *v*^1/2^.

Although the voltammetric performance of the ePAD/BCF sensor is lower compared to other disposable sensors reported in the literature [33,36,37], this sensor demonstrated a linear response when the cyclic voltammetry (CV) scan rate was varied from 10 to 100 mV s^−1^ (Figure 3A). A progressive and linear increase in redox process currents as a function of scan rate is observed, as shown in the *I*_pa_/*I*_pc_ vs. *v*^1/2^ plot (Figure 3B). The linear relationship between *I* and *v*^1/2^ (Figure 3B) indicates that the studied process is primarily diffusion-controlled, allowing the effects of capacitive charges to be neglected in this system [33].

The electroactive area of the ePAD/BCF sensor was calculated using the Randles–Sevcik equation (Equation (1)):

(1) $$I_{p}=2.69\times 10^{5}\;n^{\frac{3}{2}}\;A\;D^{\frac{1}{2}}\;C\;v^{\frac{1}{2}}$$

where *I*_p_ refers to the peak current (anodic or cathodic), *A* corresponds to the electroactive area (cm^2^), *C* is the concentration of the probe used (mol cm^−3^), *D* is the diffusion coefficient of the species in solution [Fe(CN)_6_]^3−/4−^ (7.6 × 10^−6^ cm^2^ s^−1^) and *n* refers to the number of electrons transferred in the redox reaction and *v* is the scanning speed [38,39]. Rearranging Equation (1) in terms of the electroactive area of the electrode, we can compare it with the curve equation in the graph in Figure 3B (*I*_pa_ vs. *v*^1/2^). Therefore, the first term of Equation (2) corresponds to the slope of the graph (*I*_pa_ vs. *v*^1/2^):

(2) $$A=\frac{I_{p}}{v^{\frac{1}{2}}}x\;\frac{1}{2.69\times 10^{5}\;n^{\frac{3}{2}}\;D^{\frac{1}{2}}\;C\;}$$

Considering the angular coefficient as 1.36 × 10^−3^ (after converting µA to *A*), we conclude that the electroactive area of the ePAD/BCF sensor is 0.37 cm^2^.

### 2.3. Electrochemical Behavior of Paracetamol

**Figure 4:**
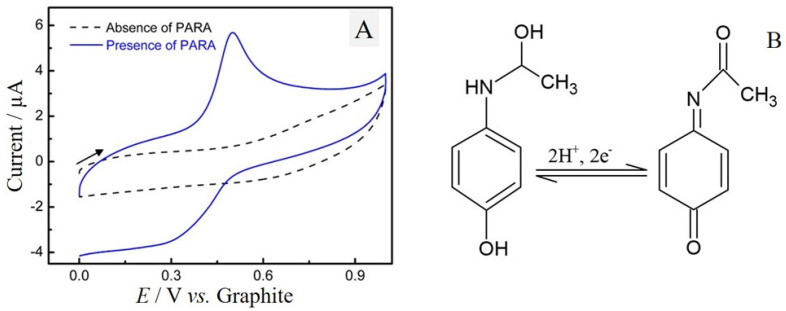
(**A**) Cyclic voltammograms of the ePAD/BCF sensor in the absence (dashed line) and presence of 200 μmol L^−1^ of PARA in 0.1 mol L^−1^ of PBS (pH 7.0), *v* = 50 mV s^−1^. (**B**) Paracetamol oxidation mechanism to N-acetyl-p-benzoquinonaimine on the ePAD/BCF sensor.

The electrochemical behavior of PARA in the ePAD/BCF sensor was evaluated by cyclic voltammetry (CV) (*v* = 50 mV s^−1^) with a volume of 150 µL of PBS 0.2 mol L^−1^ pH 7.0, containing 200 µmol L^−1^ of PARA. The results presented in Figure 4A show that no oxidation or reduction peak was observed for the voltammetric response in the absence of PARA (dashed line). However, in the presence of PARA (solid line), a reversible process is observed with an oxidation process at *E*_pa_ = 500 mV vs. graphite, and a reduction process at *E*_pc_ = 300 mV vs. graphite. Figure 4B shows the redox mechanism involved in this process, where PARA is oxidized to N-acetyl-p-benzoquinonaimine and consequently reduced back to the original molecule [40].

**Figure 5:**
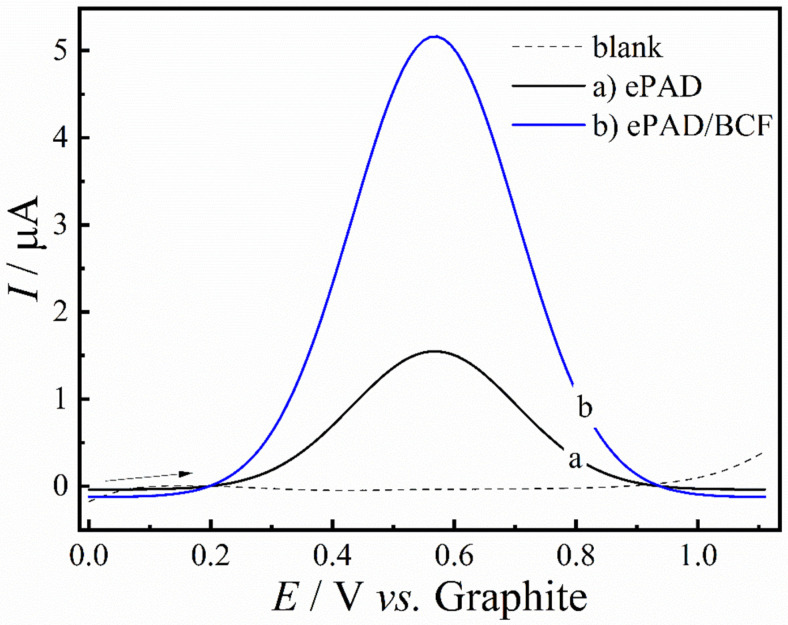
LSV voltammograms obtained with the ePAD sensor in the absence (dashed line) and in the presence (curve b) of 50 μmol L^−1^ of PARA, as well as the ePAD/BCF sensor in the presence of 50 μmol L^−1^ of PARA (curve c) (*v* = 25 mV s^−1^).

Figure 5 presents linear sweep voltammetry (LSV) voltammograms obtained for different sensors. The measurements were carried out in the potential range between 0 and 1.1 V, in 0.1 mol L^−1^ PBS pH 7.0 in the absence (dashed line) and presence of 50 µmol L^−1^ of PARA, for the ePAD sensor (curve a) and for the ePAD/BCF sensor (curve b). As illustrated in the figure, there was a significant increase in the anode peak current for the sensor modified with the BCF material, which was, therefore, selected for the construction of the calibration curve.

### 2.4. Calibration Curve

**Figure 6:**
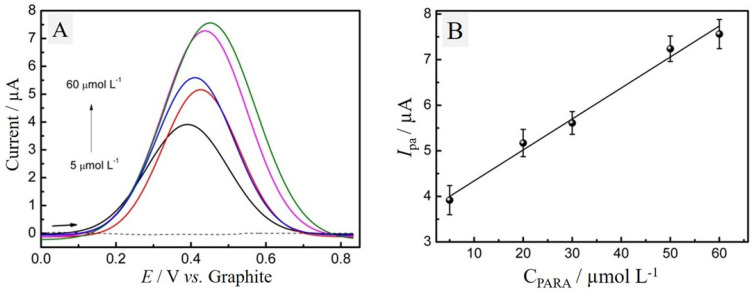
(**A**) LSV voltammograms obtained with 150 μL of 0.1 mol L^−1^ PBS solution, pH 7.0 with different concentrations of PARA ranging from 5.0 to 60 μmol L^−1^ and (**B**) linear relationship between anodic peak current and PARA concentration.

After electrochemical characterization by CV, the ePAD/BCF sensor was used to detect PARA using LSV. These experiments were conducted within the potential range of 0 to 1.1 V, with *v* = 25 mV s^−1^. Aliquots of the supporting electrolyte containing PARA concentrations of 5, 20, 30, 50 and 60 µmol L^−1^ were used, as shown in the voltammograms in Figure 6A. We observe a linear increase in the anodic peak current (*I*_pa_) as the concentration of PARA increases, ranging from 5.0 to 60 µmol L^−1^ with an R^2^ of 0.9834 (Figure 6B). The limit of detection (LOD) is 3.50 µmol L^−1^, calculated using the calibration curve method with the equation LOD = 3σ/S, where σ is the standard deviation of the blank and S is the slope of the calibration curve.

*I*_pa_ (µA) = 3.70 + 0.07 × C_PARA_ (µmol L^−1^) (3)

The slight peak potential shift observed in Figure 6A likely results from interactions between the analyte and electrode surface, as well as increased analyte concentration in the diffusion layer, which can alter electrochemical conditions and the energy required for the redox reaction [41]. Additionally, factors like double-layer capacitance and changes in the ionic strength of the supporting electrolyte may contribute to this shift.

In this study, the peak potential shift was approximately 5 mV across the concentration range tested, which is within acceptable limits for electrochemical measurements and is considered minor. Theyagarajan et al. [9] have also reported similar shifts due to analyte–electrode surface interactions at higher concentrations.

The ePAD/BCF sensor exhibited detection limits comparable to those of sensors using graphite-based inks or cost-effective binders [26,33]. Additionally, incorporating other carbon materials into the ink, such as multi-walled carbon nanotubes (MWCNTs) [36], reduced graphene nanoribbons (rGNRs) [42], and commercially available carbon inks [43], could further reduce detection limits. The ePADs also demonstrated analytical performance on par with literature-reported values for the detection of various molecules, including drugs, endocrine disruptors, and neurotransmitters, as shown in Table 2. 

The stability of the ePAD/BCF sensor was evaluated using a standard solution of 25 µM acetaminophen in a three-electrode setup. The initial current response for the 25 µM standard was 5.5 µA, and the final current was 5.32 µA, with these values representing the average response from three separate ePAD/BCF sensors. The stability of the sensor was calculated using the following formula:

(4) $$Stability\left(\%\right)=1-\frac{I_{0\;}-I_{1\;}}{I_{0\;}}$$

Substituting the measured values into this equation, the stability was found to be approximately 96.7%, indicating minimal signal degradation. This high stability suggests that the sensor retains a consistent and reliable response over repeated measurements, which is crucial for accurate quantification in practical applications. The stable current response also reflects the durability of the biochar modification, as it ensures that the sensor can withstand multiple analyses without significant loss of performance.

### 2.5. Application of the ePAD/BCF Sensor in the Quantification of Paracetamol

**Figure 7:**
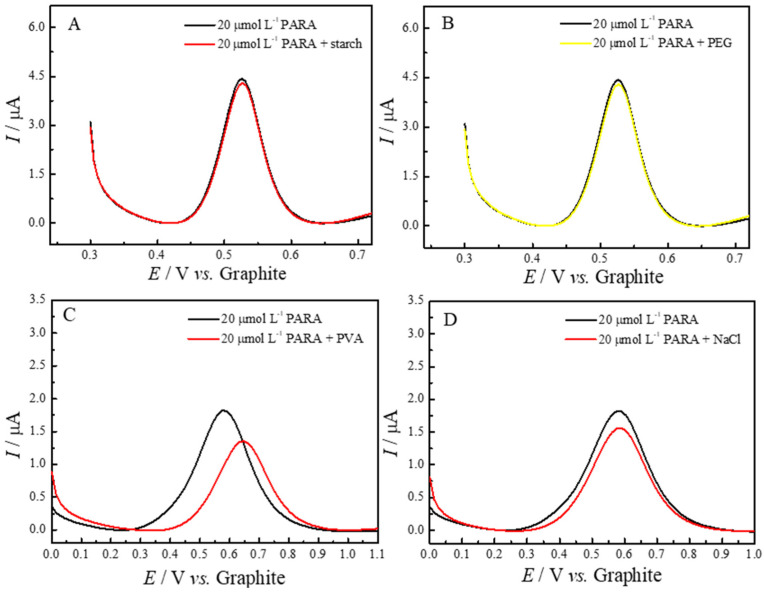
LSV voltammograms with 20 µmol L^−1^ of PARA, showing the electrochemical response before and after the addition of (**A**) starch, (**B**) PEG, (**C**) PVA and (**D**) sodium chloride (1:1 ratio). These additions assess the impact of common matrix components on PARA detection.

The respective voltammograms are shown in Figure 7, where each panel (A–D) illustrates the response of the ePAD/BCF sensor in the presence of 20.0 μmol L^−1^ PARA and one of the potential interferents: starch (A), PEG (B), PVA (C) and NaCl (D). As observed, the voltammetric profiles demonstrate minimal interference from these substances, with no significant changes in peak current or shifts in oxidation potential for most interferents. As shown in Figure 7A, the addition of starch did not produce any noticeable alteration in the peak shape or current, indicating that this common excipient does not interact significantly with the electrochemical response of paracetamol. Similarly, in Figure 7B, PEG showed no substantial effect on the peak potential or intensity, suggesting good compatibility with the sensor. For NaCl, as shown in Figure 7D, a slight decrease in peak current is observed, likely due to minor ionic interactions, but this does not affect the overall selectivity or accuracy of the sensor. The only noticeable effect is seen in Figure 7C, where the presence of PVA leads to minor variations in peak shape and intensity. Both peaks in this voltammogram are attributed to paracetamol, and this behavior is likely due to the interaction of PVA with the electrode surface, affecting the analyte’s diffusion and altering the electrochemical interface slightly. Despite these minor variations, the overall recovery of the paracetamol signal remains above 90% for all tested interferents, indicating that the ePAD/BCF sensor maintains high selectivity and reliability in detecting paracetamol even in complex sample matrices.

Table 3 summarizes the effect of potential interferents on the linear sweep voltammetry (LSV) determination of 20 μmol L^−1^ of paracetamol (PARA) at a 1:1 ratio of [PARA]/[interferent]. The results indicate minimal interference, with all tested substances causing less than a 10% change in the current response. Specifically, starch and PEG exhibited very low interference levels, at −3.20% and −5.00%, respectively, suggesting minimal interaction with the electrochemical detection of paracetamol. PVA and sodium chloride showed slightly higher interference, at −9.20% and −8.50%, respectively, which may be due to minor effects on the electrode surface or analyte diffusion. However, these variations are still within acceptable limits, as the sensor maintained over 90% of the original paracetamol signal in all cases. This demonstrates the robustness and selectivity of the ePAD/BCF sensor, confirming its suitability for accurate paracetamol quantification even in the presence of common pharmaceutical excipients and potential adulterants.

**Figure 8:**
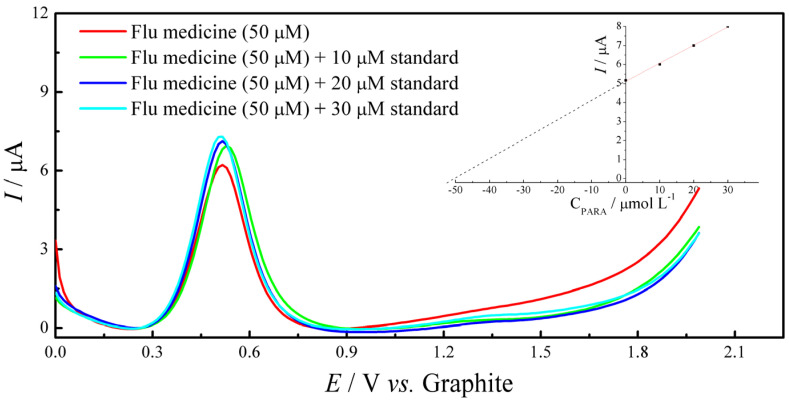
Linear sweep voltammetry (LSV) responses for a flu medicine sample with an initial concentration of 50 µM paracetamol, recorded using a graphite-based ePAD sensor. The red curve represents the LSV response of the original sample (50 µM), while subsequent standard additions of paracetamol at concentrations of 10 µM (green), 20 µM (blue) and 30 µM (cyan) show progressive increases in peak current. The inset displays a linear calibration plot of peak current (*I*_pa_) vs. C_PARA_, validating the linearity and accuracy of the standard addition method for quantifying paracetamol in complex matrices.

To assess the practical applicability of the ePAD/BCF sensor, we conducted quantification of paracetamol (PARA) in commercial analgesic and anti-flu pharmaceutical formulations. According to the manufacturers, each analgesic tablet contains 500 mg of paracetamol, while each anti-flu capsule contains a combination of 400 mg of paracetamol, 4 mg of chlorpheniramine maleate, and 4 mg of phenylephrine hydrochloride. Following sample preparation and appropriate dilution, paracetamol concentrations were determined using standard addition and recovery protocols with the ePAD/BCF sensor. Figure 8 shows linear sweep voltammetry (LSV) responses for a flu medicine sample containing an initial concentration of 50 µM of paracetamol, recorded using a graphite-based ePAD sensor. The red curve represents the original flu medicine sample (50 µM). Standard additions of paracetamol at concentrations of 10 µM (green), 20 µM (blue) and 30 µM (cyan) were subsequently added to the sample, resulting in a progressive increase in peak current for the redox process. This increase in current with each addition of standard confirms the sensor’s sensitivity and response to paracetamol concentration. The inset graph shows a linear calibration plot of peak current (*I*) versus paracetamol concentration (C_PARA_), demonstrating the linearity and quantitative capability of the standard addition method for accurate paracetamol determination in complex matrices.

Table 4 presents the labeled concentration of paracetamol, the concentration added (µmol L^−1^), the concentration quantified by the sensor (µmol L^−1^) and the relative error. All determinations were performed in triplicate to ensure the reproducibility and reliability of the results.

The ePAD/BCF sensor demonstrated satisfactory performance in quantifying paracetamol in both pharmaceutical matrices, with relative errors of −6.50% for the analgesic tablet and −7.50% for the anti-flu capsule. These deviations fall within acceptable limits for analytical applications, indicating that the sensor provides accurate and reliable measurements of paracetamol, even in the presence of additional active ingredients and excipients. The observed accuracy and selectivity underscore the ePAD/BCF sensor’s potential as a practical tool for the quality control of paracetamol in complex drug formulations.

## 3. Materials and Methods

### 3.1. Material and Reagents

Paracetamol (acetaminophen), Potassium Chloride (KCl), Potassium phosphate monobasic (KH_2_PO_4_), Sodium phosphate dibasic (Na_2_HPO_4_), Potassium ferricyanide (III) (K_3_[Fe(CN)_6_]), Potassium ferrocyanide (II) (K_4_[Fe(CN)_6_]), starch (C_6_H_10_O_5_)n), sodium chloride (NaCl), graphite powder, Poly(ethylene glycol) (PEG), and Poly(vinyl alcohol) were purchased from Sigma Aldrich (St. Louis, MO, USA) and were of analytical grade. Glass varnish (Acrilex^®^, São Bernardo do Campo, SP, Brazil) was obtained from a local stationery store. 

Mohini Sain of the University of Toronto generously donated the bamboo-based biochar (BC) used in this work, which was produced by pyrolyzing bamboo biomass. For functionalization, 1 g of BC was kept for 14 h in 500 mL of a mixture of H_2_SO_4_/HNO_3_ in a 3:1 ratio. Then, the mixture with BC was filtered by a 0.45 µm GVS nylon membrane. The material that remained on the membrane was dried in an oven set to 60 °C after being cleaned with ultrapure water until the pH reached neutral. The resulting material was the BCF, and a stock solution of 1.0 mg/mL was prepared in ultrapure water and kept under refrigeration.

### 3.2. Instruments

Cyclic voltammetry (CV) and linear sweep voltammetry (LSV) were carried out using a 6005E electrochemical workstation (CH Instruments Inc., Austin, TX, USA) equipped with Chi6005e software (CHI version 16.08). The morphological characterization was performed by scanning electron microscopy using FEI NovaNanoSEM 450 (Thermo Fisher Scientific, Waltham, MA, USA).

### 3.3. Fabrication and Modification of ePADs 

**Figure 9:**
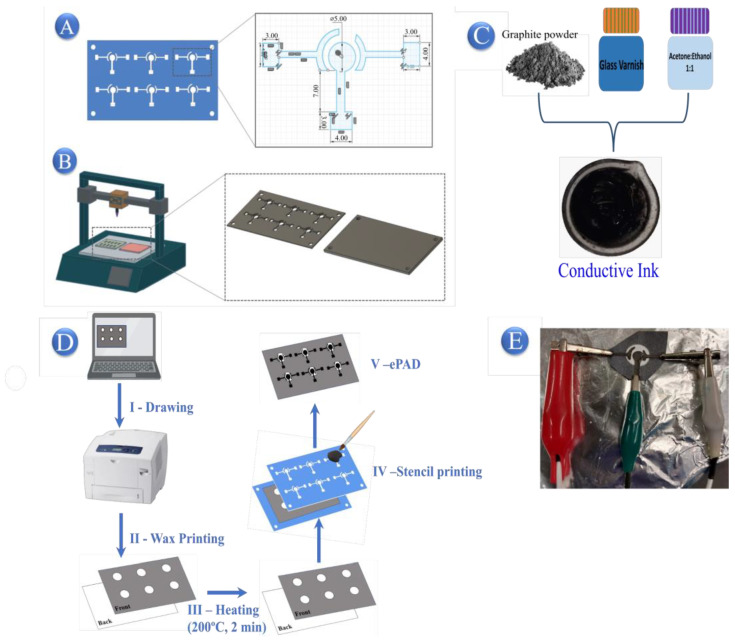
Sequential fabrication of ePADs: (**A**) Stencil design conceptualized in PLA for sensor creation. (**B**) Stencil produced using PLA filament via 3D printing. (**C**) Preparation of conductive ink. (**D**) Definition of the electrolyte area, including the following steps: (I) digital design, (II) wax printing, (III) wax melting to create a hydrophobic barrier within the paper, (IV) sensor fabrication using the stencil and conductive ink and (V) final assembly of the ePAD. (**E**) Setup adjustment for electrochemical measurements with metallic clips.

The stencil was specifically designed to facilitate the printing of six sensors in each batch. It is composed of two main components: a 3D plate featuring two rows of three electrodes (working, reference and auxiliary) and a support plate for the printing process. These two components are connected using butterfly screws, as illustrated in Figure 9A,B. The stencil was manufactured utilizing fused deposition modeling technology, employing Cherry (Edison, NJ, USA) EasyFill 1.75mm Polylactic Acid (PLA) filament. The printing process was carried out using an ANET ET4-PRO printer (Shenzhen, China), adhering to precise conditions such as a nozzle temperature of 200 °C, a bed temperature of 65 °C and a layer thickness of 0.1 mm. The G-CODE was generated using Ultimaker Cura 4.8.0 software.

Based on the study by Pradela-Filho et al. [37], the conductive ink was formulated by mixing graphite powder and Glass varnish in a ratio of 60% to 40%, along with 400 µL of an ethanol/acetone mix to achieve the desired viscosity (Figure 9C).

The fabrication process of ePAD sensors is illustrated in Figure 9D. To begin, a hydrophobic pattern was created to establish an electrolyte reservoir for voltammetry analysis. Using Inkscape software (version 1.3.2), black dots on a white background were designed and printed onto paper using a Xerox ColorQube 8580 printer (Norwalk, CT, USA). Subsequently, the wax paper was heated in an oven at 100 °C for 2 min to generate a hydrophobic barrier surrounding the hydrophilic region. Lastly, the three-electrode system was printed onto this setup utilizing the PLA stencil, and conductive ink was applied with a brush.

Finally, the ePADs were prepared for voltammetric experiments. The sensors were dried at room temperature for 12 h before use. Each ePAD measured approximately 2.5 cm in height and width, with a working electrode (WE) diameter of 5 mm. Approximately 150 mg of conductive ink was used per manufacturing batch. Prior to the voltammetric experiments, the ePAD working electrode was modified with 10 μL of a BCF suspension at a concentration of 1.0 mg/mL. Figure 9E illustrates the setup adaptations for analysis, using metallic clips to secure the ePAD.

### 3.4. Sample Preparation

A stock solution of standard PARA at a concentration of 1 mmol L^−1^ was prepared for the purpose of characterization and comparison analysis. To quantify PARA, tablets containing 500 mg of paracetamol and anti-flu capsules containing 400 mg of paracetamol, 4 mg of chlorpheniramine maleate and 4 mg of phenylephrine hydrochloride were obtained from a local pharmacy. The samples underwent maceration and weighing procedures. Subsequently, two theoretical standard solutions were created with a concentration of 20.0 μmol L^−1^ of PARA, utilizing a 0.1 mol L^−1^ PBS solution at pH 7. The quantification of PARA in the samples was determined by experiments of addition and recovery.

## 4. Conclusions

This study introduces a novel approach for fabricating electrochemical paper-based analytical devices (ePADs) using a 60:40 (m/m) graphite-to-glass varnish (GR-GV) conductive ink and a sustainable functionalized bamboo-derived biochar (BCF) modification. The integration of BCF significantly enhanced the sensor’s voltammetric response, particularly with the redox probe [Fe(CN)_6_]^3−/4−^, demonstrating improved electron transfer and an electroactive area of 0.37 cm^2^, which contributes to its high sensitivity. The ePAD/BCF sensor exhibited a linear detection range for paracetamol (PARA) from 5 to 60 μmol L^−1^, with a detection limit of 3.50 μmol L^−1^, enabling low-concentration detection.

Practical applicability was confirmed through successful quantification of PARA in commercial tablets and anti-flu capsules, achieving recoveries of 92.50% and 93.50%, respectively, even in complex matrices. Additionally, the sensor showed strong selectivity against common interferents, underscoring its potential for identifying counterfeit or substandard drugs. These results position the ePAD/BCF sensor as a promising, accessible and cost-effective tool for electrochemical analysis in pharmaceutical quality control and public health applications, offering an innovative and sustainable approach for accurate substance detection.

## Figures and Tables

**Table 1:** Optimization of the ePAD sensor manufacturing process using different conductive ink compositions and modification with BCF material. Comparison of anodic peak currents (*I*_pa_), cathodic peak currents (*I*_pc_), and redox peak separation (Δ*E*_p_).

Scheme	GR-GV Ratio	*I*_pa_ (µA)	*I*_pc_ (µA)	Δ*E*_p_ (mV)
	Ink 01—80:20 (m/m)	N/A	N/A	N/A
ePAD	Ink 02—70:30 (m/m)	158	−159	1420
	Ink 03—60:40 (m/m)	213	−208	772
	Ink 04—50:50 (m/m)	187	−190	980
ePAD/BCF		294	−284	387

**Table 2:** Characteristics of paper-based electrochemical analytical devices.

Manufacturing Method	Conductive Ink	Analyte	LOD (µmol L^−1^)	Ref.
Spread with brush	Graphite/nail polish 80:20% (m/m)	Dopamine	5.2	[37]
Adhesive mask	Graphite/glass varnish 80:20% (m:m)	Dopamine	4.1	[33]
		Catechol	9.0	
		Hydroquinone	5.3	
		Estriol	0.08	
	Graphite/nail polish 52:48% (m/m) + 20 µL of MWCNT suspension	Caffeic acid	0.2	[36]
Screen-printed	Graphite-based ink and Ag/AgCl ink	Bisphenol-A	0.03	[44]
	Carbon powder (10% m/m) with 9.0 g of graphite ink	Capsaicin	0.085	[43]
	Carbon ink and rGNRs	Sulfamethoxazole	0.09	[42]
		Trimethoprim	0.04	
3D-printed stencil	GR-GV 60:40% (m/m) + 400 μL acetone/ethanol (1:1)	PARA	3.50	*

* this work.

**Table 3:** Effect of potential interferents on the LSV determination of 20 μmol L^−1^ paracetamol (PARA) at a 1:1 [PARA]/[interferent] ratio.

Interferents	Interference (%)
Starch	−3.20
PEG	−5.00
PVA	−9.20
Sodium Chloride	−8.50

**Table 4:** Determination of paracetamol (PARA) in real samples of commercial paracetamol tablets and anti-flu capsules. The table presents the labeled values in mg for each drug, the added concentration (µmol L^−1^), the concentration quantified by the ePAD/BCF sensor, the recovery percentage (%) and the standard error of recovery (%).

Samples Primary Ingredients	Labeled (mg)	Added (µmol L^−1^)	Quantified (µmol L^−1^)	Error (µmol L^−1^)	Error (%)	Recovery (%)	Standard Error of Recovery (%)
Paracetamol Tablet	500 mg/tablet	20.00	18.70 ± 0.03	0.03	−6.50%	93.50	0.15
Flu medicine capsule	Paracetamol 400 mg Chlorpheniramine Maleate 4 mg Phenylephrine Hydrochloride 4 mg	20.00	18.50 ± 0.05	0.05	−7.50%	92.50	0.25

## Data Availability

Data are available from the authors on reasonable request.

## References

[1] Roncone A., Kelly S.D., Giannioti Z., Hauk C., Caillet C., Newton P.N., Perez-Mon C., Bontempo L. (2024). Stable Isotope Ratio Analysis: An Emerging Tool to Trace the Origin of Falsified Medicines. TrAC Trends Anal. Chem..

[2] Sandeep D.H., Krushna B.R.R., Sharma S.C., Ravindran P., Sivayogana R., Ramesha H., Hemalatha N., Rashmi H., Devaraju K.S., Krithika C. (2024). Eco-Friendly Synthesis of CQDs from Pistachio Shells: Versatile Applications in Anti-Counterfeiting, Flexible Films, Latent Fingerprints and Potential Anti-Cancer Activity. J. Alloys Compd..

[3] Pal T., Mathai T., Mukherji S. (2023). Colorimetric Chemosensor for Rapid Detection of Fluoroquinolone Load in Environmental Water Bodies, Urine, and Counterfeit Drug Testing. Biosens. Bioelectron. X.

[4] Mazurków J.M., Montiel N.F., Van Echelpoel R., Kusior A., De Wael K. (2024). The Potential of Electrochemical Sensors to Unveil Counterfeits: Xanax as a Case Study. Electrochim. Acta.

[5] Mohammad M.A.A., Elkady E.F., Fouad M.A., Salem W.A. (2021). Analysis of Aspirin, Prasugrel and Clopidogrel in Counterfeit Pharmaceutical and Herbal Products: Plackett-Burman Screening and Box-Behnken Optimization. J. Chromatogr. Sci..

[6] Schwartzman G.H., Dekker P.K., Silverstein A.S., Fontecilla N.M., Norton S.A. (2022). Dermatologic Consequences of Substandard, Spurious, Falsely Labeled, Falsified, and Counterfeit Medications. Dermatol. Clin..

[7] Vandy A., Conteh E., Lahai M., Kolipha-Kamara M., Marah M., Marah F., Suma K.M., Mattia S.C., Tucker K.D.S., Wray V.S.E. (2024). Physicochemical Quality Assessment of Various Brands of Paracetamol Tablets Sold in Freetown Municipality. Heliyon.

[8] Achache M., Elouilali Idrissi G., Chraka A., Ben Seddik N., Draoui K., Bouchta D., Mohamed C. (2024). Detection of Paracetamol by a Montmorillonite-Modified Carbon Paste Sensor: A Study Combining MC Simulation, DFT Computation and Electrochemical Investigations. Talanta.

[9] Theyagarajan K., Lakshmi B.A., Kim Y.J. (2024). Electrochemical Sensing of Acetaminophen in Biofluids, Pharmaceutical and Environmental Samples Using Cobalt Hexacyanoferrate Decorated Iron Terephthalate Metal Organic Framework. Electrochim. Acta.

[10] Omar J., Boix A., Ulberth F. (2020). Raman Spectroscopy for Quality Control and Detection of Substandard Painkillers. Vib. Spectrosc..

[11] de Sá B.S., Stefano J.S., Luiz L.R., Perfecto T.M., Mazon T., Volanti D.P., Janegitz B.C., Ribeiro C. (2024). Methane-Derived Electrochemical Sensor for Determination of Paracetamol and Diquat. Mater. Chem. Phys..

[12] Keizers P.H.J., Bakker F., Ferreira J., Wackers P.F.K., van Kollenburg D., van der Aa E., van Beers A. (2020). Benchtop NMR Spectroscopy in the Analysis of Substandard and Falsified Medicines as Well as Illegal Drugs. J. Pharm. Biomed. Anal..

[13] Sakuda M., Yoshida N., Koide T., Keila T., Kimura K., Tsuboi H. (2021). Clarification of the Internal Structure and Factors of Poor Dissolution of Substandard Roxithromycin Tablets by Near-Infrared Chemical Imaging. Int. J. Pharm..

[14] Melaré A.G., Barreto F.C., Silva M.K.L., Simões R.P., Cesarino I. (2023). Determination of Fluoxetine in Weight Loss Herbal Medicine Using an Electrochemical Sensor Based on RGO-CuNPs. Molecules.

[15] Vazan M., Tashkhourian J., Haghighi B. (2023). A Novel Electrochemical Sensor Based on MoO_3_ Nanobelt-Graphene Oxide Composite for the Simultaneous Determination of Paracetamol and 4-Aminophenol. Diam. Relat. Mater..

[16] Silva L.R.G., Stefano J.S., Crapnell R.D., Banks C.E., Janegitz B.C. (2023). Additive Manufactured Microfluidic Device for Electrochemical Detection of Carbendazim in Honey Samples. Talanta Open.

[17] Zhang W., Sun Q., Zhang X., Yuan W., Wu J. (2023). A Laser-Induced Graphene-Based Sensor Modified with CeO_2_ for Determination of Organophosphorus Pesticides with Improved Performance. Sensors.

[18] Thakur A., Kumar A. (2023). Exploring the Potential of Ionic Liquid-Based Electrochemical Biosensors for Real-Time Biomolecule Monitoring in Pharmaceutical Applications: From Lab to Life. Results Eng..

[19] Hu J., Dai J., Huang C., Zeng X., Wei W., Wang Z., Lin P. (2023). Organic Electrochemical Transistor with MoS_2_ Nanosheets Modified Gate Electrode for Sensitive Glucose Sensing. Sensors.

[20] Eldeeb M.A., Dhamu V.N., Paul A., Muthukumar S., Prasad S. (2023). Espial: Electrochemical Soil PH Sensor for In Situ Real-Time Monitoring. Micromachines.

[21] Wang Z., Shi Y., Xu Z., Sun M., Shen X., Wu K., Yu M., Zhang L., Yu G. (2024). Development of a C3N4-Based Electrochemical Sensing Platform for the Detection of Circulating Tumor Cells in Blood. Alex. Eng. J..

[22] Barreto F.C., dos Santos G.T.V., Leao A.L., Goonetilleke A., Cesarino I. (2024). Determination and Electro-Remediation of Sulfamethazine Using Carbon Nanotubes and Silver Nanoparticles as Electrode Modifiers. J. Solid State Electrochem..

[23] Cancelliere R., Cianciaruso M., Carbone K., Micheli L. (2022). Biochar: A Sustainable Alternative in the Development of Electrochemical Printed Platforms. Chemosensors.

[24] Barreto F.C., Ito E.Y., Mounienguet N.K., Dal’ Evedove Soares L., Yang J., He Q., Cesarino I. (2023). Electrochemical Sensor Based on Spent Coffee Grounds Hydrochar and Metal Nanoparticles for Simultaneous Detection of Emerging Contaminants in Natural Water. Chemosensors.

[25] Hassan Q., Meng Z., Noroozifar M., Kerman K. (2023). Methylene Blue-Modified Biochar from Sugarcane for the Simultaneous Electrochemical Detection of Four DNA Bases. Chemosensors.

[26] Gomes G.C., da Silva M.K.L., Barreto F.C., Cesarino I. (2023). Electrochemical Sensing Platform Based on Renewable Carbon Modified with Antimony Nanoparticles for Methylparaben Detection in Personal Care Products. Chemosensors.

[27] Baharfar M., Rahbar M., Tajik M., Liu G. (2020). Engineering Strategies for Enhancing the Performance of Electrochemical Paper-Based Analytical Devices. Biosens. Bioelectron..

[28] Silva M.K.L., Sousa G.S., Simoes R.P., Cesarino I. (2022). Fabrication of Paper-Based Analytical Devices Using a PLA 3D-Printed Stencil for Electrochemical Determination of Chloroquine and Escitalopram. J. Solid State Electrochem..

[29] Mustafa F., Finny A.S., Kirk K.A., Andreescu S. (2020). Printed Paper-Based (Bio)Sensors: Design, Fabrication and Applications. Compr. Anal. Chem..

[30] Squissato A.L., Almeida E.S., Silva S.G., Richter E.M., Batista A.D., Munoz R.A.A. (2018). Screen-Printed Electrodes for Quality Control of Liquid (Bio)Fuels. TrAC Trends Anal. Chem..

[31] Nunes E.W., Silva M.K.L., Rascón J., Leiva-Tafur D., Lapa R.M.L., Cesarino I. (2022). Acetylcholinesterase Biosensor Based on Functionalized Renewable Carbon Platform for Detection of Carbaryl in Food. Biosensors.

[32] Kalinke C., Oliveira P.R., Oliveira G.A., Mangrich A.S., Marcolino-Junior L.H., Bergamini M.F. (2017). Activated Biochar: Preparation, Characterization and Electroanalytical Application in an Alternative Strategy of Nickel Determination. Anal. Chim. Acta.

[33] Pradela-Filho L.A., Andreotti I.A.A., Carvalho J.H.S., Araújo D.A.G., Orzari L.O., Gatti A., Takeuchi R.M., Santos A.L., Janegitz B.C. (2020). Glass Varnish-Based Carbon Conductive Ink: A New Way to Produce Disposable Electrochemical Sensors. Sensors Actuators B Chem..

[34] Allende S., Liu Y., Jacob M.V. (2024). Electrochemical Sensing of Paracetamol Based on Sugarcane Bagasse-Activated Biochar. Ind. Crops Prod..

[35] Zeghioud H., Fryda L., Djelal H., Assadi A., Kane A. (2022). A Comprehensive Review of Biochar in Removal of Organic Pollutants from Wastewater: Characterization, Toxicity, Activation/Functionalization and Influencing Treatment Factors. J. Water Process Eng..

[36] Araújo D.A.G., Camargo J.R., Pradela-Filho L.A., Lima A.P., Muñoz R.A.A., Takeuchi R.M., Janegitz B.C., Santos A.L. (2020). A Lab-Made Screen-Printed Electrode as a Platform to Study the Effect of the Size and Functionalization of Carbon Nanotubes on the Voltammetric Determination of Caffeic Acid. Microchem. J..

[37] Pradela-Filho L.A., Araújo D.A.G., Takeuchi R.M., Santos A.L. (2017). Nail Polish and Carbon Powder: An Attractive Mixture to Prepare Paper-Based Electrodes. Electrochim. Acta.

[38] Crapnell R.D., Banks C.E. (2021). Perspective: What Constitutes a Quality Paper in Electroanalysis?. Talanta Open.

[39] Ferrari A.G.M., Foster C.W., Kelly P.J., Brownson D.A.C., Banks C.E. (2018). Determination of the Electrochemical Area of Screen-Printed Electrochemical Sensing Platforms. Biosensors.

[40] Camargo J.R., Andreotti I.A.A., Kalinke C., Henrique J.M., Bonacin J.A., Janegitz B.C. (2020). Waterproof Paper as a New Substrate to Construct a Disposable Sensor for the Electrochemical Determination of Paracetamol and Melatonin. Talanta.

[41] Bard A.J., Faulkner L.R. (2001). Electrochemical Methods: Fundamentals and Applications.

[42] Martins T.S., Bott-Neto J.L., Oliveira O.N., Machado S.A.S. (2021). Paper-Based Electrochemical Sensors with Reduced Graphene Nanoribbons for Simultaneous Detection of Sulfamethoxazole and Trimethoprim in Water Samples. J. Electroanal. Chem..

[43] Deroco P.B., Fatibello-Filho O., Arduini F., Moscone D. (2020). Electrochemical Determination of Capsaicin in Pepper Samples Using Sustainable Paper-Based Screen-Printed Bulk Modified with Carbon Black. Electrochim. Acta.

[44] Jemmeli D., Marcoccio E., Moscone D., Dridi C., Arduini F. (2020). Highly Sensitive Paper-Based Electrochemical Sensor for Reagent Free Detection of Bisphenol A. Talanta.

